# N-6 Polyunsaturated Fatty Acids and Risk of Cancer: Accumulating Evidence from Prospective Studies

**DOI:** 10.3390/nu12092523

**Published:** 2020-08-20

**Authors:** Youngyo Kim, Jeongseon Kim

**Affiliations:** 1 Department of Food and Nutrition/Institute of Agriculture and Life Science, Gyeongsang National University, Jinju 52828, Korea; youngyokim@gnu.ac.kr; 2 Department of Cancer Biomedical Science, Graduate School of Cancer Science and Policy, National Cancer Center, Goyang 10408, Korea

**Keywords:** n-6 fatty acids, cancer, meta-analysis, prospective studies

## Abstract

Previous studies on the association between polyunsaturated fatty acids (PUFAs) and cancer have focused on n-3 PUFAs. To investigate the association between intake or blood levels of n-6 PUFAs and cancer, we searched the PubMed and Embase databases up to March 2020 and conducted a meta-analysis. A total of 70 articles were identified. High blood levels of n-6 PUFAs were associated with an 8% lower risk of all cancers (relative risk (RR) = 0.92; 95% confidence interval (CI): 0.86–0.98) compared to low blood levels of n-6 PUFAs. In the subgroup analyses by cancer site, type of n-6 PUFAs, and sex, the inverse associations were strong for breast cancer (RR = 0.87; 95% CI: 0.77–0.98), linoleic acid (LA) (RR = 0.91; 95% CI: 0.82–1.00), and women (RR = 0.88; 95% CI: 0.79–0.97). In the dose-response analysis, a 2% and 3% decrease in the risk of cancer was observed with a 5% increase in blood levels of n-6 PUFAs and LA, respectively. Thus, there was no significant association between n-6 PUFA intake and the risk of cancer. The pooled RR of cancer for the highest versus lowest category of n-6 PUFA intake was 1.02 (95% CI: 0.99–1.05). Evidence from prospective studies indicated that intake of n-6 PUFAs was not significantly associated with risk of cancer, but blood levels of n-6 PUFAs were inversely associated with risk of cancer.

## 1. Introduction

Polyunsaturated fatty acids (PUFAs) are long-chain fatty acids which include more than one double bond in their backbone and are classified as n-3 PUFAs and n-6 PUFAs, according to the position of the first double bond in the carbon chain [1]. Generally, PUFAs have been considered beneficial for human health because of their anti-inflammatory effects compared to other types of fatty acids, such as saturated or trans fatty acids [2]. However, existing evidence for the association between PUFAs and chronic disease has focused mostly on n-3 PUFAs [3]. The potential effect of n-6 PUFAs has received less attention, and results on the associations between n-6 PUFAs and disease risk have additionally been reported in studies focused on other types of fatty acids. Since high proportions of dietary n-6 PUFAs are found in the Western diet, which is spreading across the world [4], it is important to identify the association between n-6 PUFA intake and the risk of chronic disease, such as cancer.

Some studies have recommended reducing n-6 PUFA intake, pointing out that n-6 PUFAs, especially arachidonic acids (AAs), produce eicosanoids that can increase inflammatory disorders [2,5]. Most of the studies investigating the association between intake or blood levels of fatty acids and the risk of cancer have provided results by type of fatty acids, including n-6 PUFAs; however, to our knowledge, there has been no comprehensive meta-analysis exploring the association between n-6 PUFA intake and risk of all cancers since 1998 [6]. The meta-analysis published in 1998 found no evidence suggesting that high linoleic acid (LA) intake increases the risk of cancer [6]. A review evaluating the association between AA and the risk of cancer also concluded that AA was not associated with an increased risk of breast or prostate cancer [7]. It is not easy to compute the intake of specific fatty acids accurately [8], and misclassification often occurs [9]. Regarding PUFAs, the measurement of biomarkers may provide a more accurate assessment of intake because PUFAs are rarely endogenously synthesized [10]. Therefore, it is necessary to consider both intake and biomarkers of fatty acids when analyzing the association between PUFAs and the risk of disease. To clarify the association between the intake or blood levels of n-6 PUFAs and the risk of cancer, we conducted a systematic review and meta-analysis of prospective studies.

## 2. Materials and Methods

According to the Preferred Reporting Items for Systematic Reviews and Meta-Analyses (PRISMA) statement [11], two authors (Y.K. and J.K.) independently performed a literature search, study selection, data extraction, and quality assessment. Any discrepancies were addressed through reviewing the original articles and discussion.

### 2.1. Literature Search and Study Selection

We searched for eligible articles published up to March 2020 in the PubMed and Embase databases. The search terms were as follows: “(fat OR fatty acids) AND (cancer) AND (risk OR incidence)”. To supplement the search, we reviewed the reference lists of previous reviews and the retrieved articles.

To be included in this meta-analysis, articles had to meet the following criteria: (1) prospective design (cohort or nested case-control); (2) intake of n-6 PUFAs or blood levels of n-6 PUFAs as the exposure of interest; (3) cancer as the outcome of interest; and (4) reporting of relative risks (RRs) and 95% confidence intervals (CIs). If more than one article provided the RRs for risk of the same cancer site from the same cohort, the study including the largest population was selected for the meta-analysis.

### 2.2. Data Extraction

The following information was extracted: first author’s surname, publication year, region of study, cohort name, dates of study period, sex and baseline age of subjects, number of cases and subjects/controls or person-time, types of cancer, types of n-6 PUFAs, RRs with corresponding 95% CIs for all categories of n-6 PUFA intake or blood levels of n-6 PUFAs, and adjustment factors. We included the RRs that were maximally adjusted for potential confounders in the meta-analysis.

### 2.3. Quality Assessment

The study quality was evaluated using the Newcastle-Ottawa quality assessment scale [12]. This scale is based on 3 domains: selection of participants, comparability of study groups, and ascertainment of outcomes of interest. Studies with scores of 6 or less, 7 to 9, and 10 or higher (out of 13) were considered low, good, and high quality, respectively.

### 2.4. Statistical Analysis

The pooled RRs and 95% CIs were calculated using the DerSimonian and Laird random-effects model [13]. Any results provided separately by sex, cancer stage, specific tumor sites (e.g., colon or rectal), or types of n-6 PUFAs were combined using a fixed-effects model and included in the main analysis. The separately reported RRs were used in the subgroup analysis by sex or types of n-6 PUFAs. We performed subgroup analyses by cancer site, type of n-6 PUFAs, and sex when data were available. When one article reported RRs of both all cancers and type of cancer (e.g., breast or prostate), we included RRs of all cancers in the main analysis. The RRs of type of cancer were used in the analysis by cancer site.

A dose-response meta-analysis was carried out using the method described by Greenland and Longnecker [14,15,16]. The median or mean value for each exposure category (n-6 PUFA intake or blood levels of n-6 PUFAs) was assigned to the corresponding RR. We assumed that open-ended categories would have the same interval as the adjacent category. Furthermore, when n-6 PUFA intake was reported as density (g/kcal or %/kcal), we converted these values to absolute intake (g/day) using the mean daily energy intake of the study participants, considering that fat provides nine calories per gram. We assessed a potential nonlinear relationship by means of restricted cubic splines with 3 knots at fixed percentiles (10%, 50%, and 90%) of the aggregated exposure. A *P* value for nonlinearity was obtained by testing the regression coefficient of the second spline equal to zero [17].

Heterogeneity across studies was assessed using the *Q* statistic [18] and quantified by the *I*^2^ statistic [19]. To explore the robustness of the results, a sensitivity analysis that excluded one study at a time and pooled the remaining studies was conducted. Begg’s [20] and Egger’s [21] tests were performed to evaluate a publication bias. The α level of statistical significance was set at *p* ≤ 0.05. All statistical analyses were conducted with STATA software version 14.2 (StataCorp, College Station, TX, USA).

## 3. Results

### 3.1. Study Characteristics

A total of 70 publications were eligible for meta-analysis of the prospective association between intake or blood levels of n-6 PUFAs and the risk of cancer [22,23,24,25,26,27,28,29,30,31,32,33,34,35,36,37,38,39,40,41,42,43,44,45,46,47,48,49,50,51,52,53,54,55,56,57,58,59,60,61,62,63,64,65,66,67,68,69,70,71,72,73,74,75,76,77,78,79,80,81,82,83,84,85,86,87,88,89,90,91]. The detailed process of study selection is presented in Supplementary Figure S1. Forty-one articles reported RRs for n-6 PUFA intake only [22,23,25,29,30,31,33,34,36,37,40,44,45,46,48,49,50,53,54,57,58,59,60,63,64,65,66,69,72,75,77,78,79,80,81,83,85,86,87,90,91], 24 articles reported RRs for blood n-6 PUFA levels only [24,26,27,28,38,39,41,42,47,51,52,55,56,61,62,67,68,70,71,74,82,84,88,89], and 5 articles provided RRs for both n-6 PUFA intake and blood n-6 PUFA levels [32,35,43,73,76]. The types of cancer could be summarized as follows: breast (23 articles and 17,546 cases) [22,26,27,32,34,38,45,46,50,54,55,62,69,71,74,77,78,79,81,82,84,87,88], prostate (17 articles and 39,038 cases) [22,40,41,42,43,52,56,61,64,65,66,68,72,73,76,86,89], colorectal (14 articles and 10,541 cases) [28,29,33,35,37,48,49,57,59,60,63,67,70,91], pancreas (5 articles and 2403 cases) [24,53,58,75,80], endometrium (3 articles and 1756 cases) [31,36,85], skin (3 articles and 36,819 cases) [23,39,44], and others (ovarian, lung, liver, lymphoma, and gastric; 6 articles and 2958 cases) [25,30,47,51,83,90]. Three articles provided RRs of any type of cancer [22,38,73]. All studies scored higher than 9 points on the quality assessment, indicating good quality, and the median value was 11 points.

### 3.2. N-6 PUFA Intake

**Figure 1:**
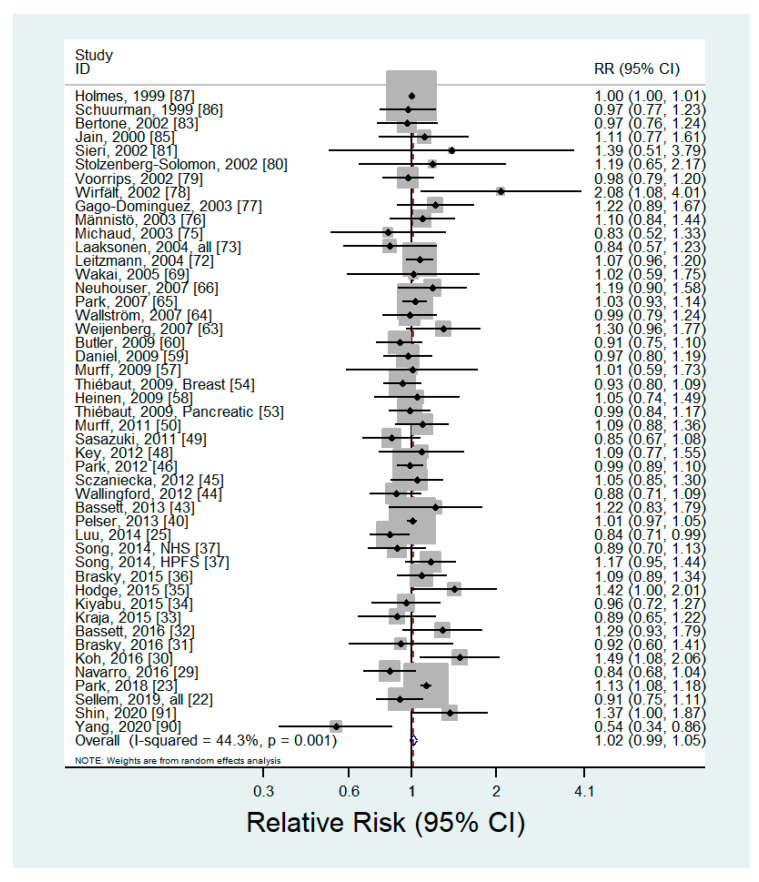
Forest plot of prospective studies of cancer for the highest versus lowest category of n-6 PUFA intake, using a random-effects model.

Forty-six articles including 99,877 cancer cases investigated the association between n-6 PUFA intake and the risk of cancer [22,23,25,29,30,31,32,33,34,35,36,37,40,43,44,45,46,48,49,50,53,54,57,58,59,60,63,64,65,66,69,72,73,75,76,77,78,79,80,81,83,85,86,87,90,91] (Supplementary Table S1). The pooled RR of any type of cancer for the highest *versus* lowest categories of n-6 PUFA intake was 1.02 (95% CI: 0.99–1.05) (Figure 1). No evidence of publication bias was found by Begg’s test (*p* = 0.15) or Egger’s test (*p* = 0.24). We did not find any significant associations in the subgroup analyses by cancer site, type of n-6 PUFAs, or sex (Table 1). In the dose-response analysis, there was no significant linear (*p* = 0.06) or nonlinear (*p* = 0.92) association between n-6 PUFA intake and the risk of cancer.

### 3.3. Blood Levels of n-6 PUFAs

**Figure 2:**
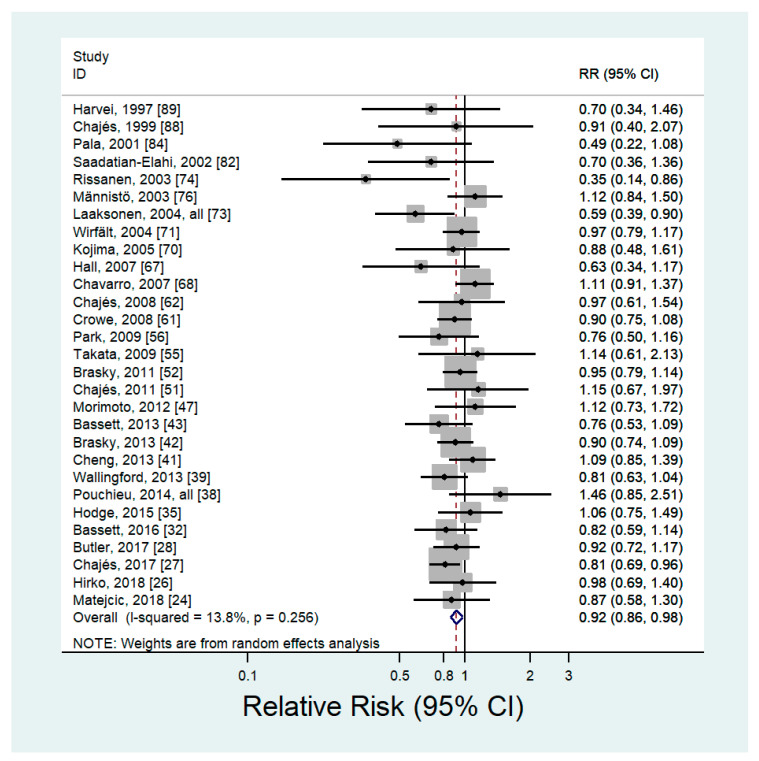
Forest plot of prospective studies of cancer for the highest versus lowest category of blood levels of n-6 PUFAs, using a random-effects model.

Twenty-nine articles including 13,966 cancer cases evaluated the association between blood levels of n-6 PUFAs and the risk of cancer [24,26,27,28,32,35,38,39,41,42,43,47,51,52,55,56,61,62,67,68,70,71,73,74,76,82,84,88,89] (Supplementary Table S2). The pooled RR of any type of cancer for the highest *versus* lowest categories of blood n-6 PUFAs was 0.92 (95% CI: 0.86–0.98) with no significant heterogeneity (*p* = 0.26, *I*^2^ = 13.8%) (Figure 2). The results of the sensitivity analysis, which removed one study at a time, showed that the pooled RR ranged from 0.90 (95% CI: 0.85–0.96) to 0.93 (95% CI: 0.87–0.99). There was no evidence of publication bias (Begg’s *P* = 0.18; Egger’s *P* = 0.22). Subgroup analysis by cancer site showed a significant inverse association between blood levels of n-6 PUFAs and breast cancer (RR = 0.87; 95% CI: 0.77–0.98), and nonsignificant inverse associations were observed between blood levels of n-6 PUFAs and prostate (RR = 0.94; 95% CI: 0.84–1.05), colorectal (RR = 0.92; 95% CI: 0.77–1.10), and other cancers (RR = 0.90; 95% CI: 0.75–1.08) (Table 2). For the type of n-6 PUFAs, the blood levels of LA showed an inverse association with the risk of cancer (RR = 0.91; 95% CI: 0.82–1.00), but other types of n-6 PUFAs indicated nonsignificant inverse associations. When analyzed by sex, a slightly stronger inverse association was observed in women (RR = 0.88; 95% CI: 0.79–0.97) than in men (RR = 0.92; 95% CI: 0.83–1.02).

**Figure 3:**
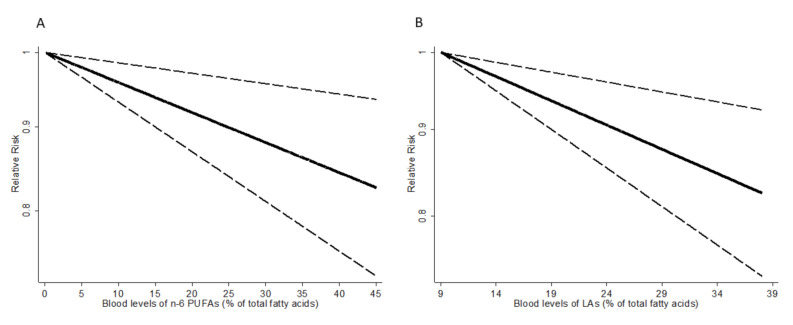
Dose-response association between blood levels of n-6 PUFAs (**A**), LA (**B**) and relative risk of cancer. The vertical axis is on a log scale.

The results of dose-response analysis showed an inverse linear association between blood levels of n-6 PUFAs and the risk of cancer. A 5% increase in blood levels of n-6 PUFAs was associated with a 2% lower risk of cancer (RR = 0.98; 95% CI: 0.97–0.99) (Figure 3A). There was no evidence of a nonlinear association (*p* = 0.43). By type of n-6 PUFAs, blood levels of LA were also inversely associated with risk of cancer (RR for 5% increase in LA = 0.97; 95% CI: 0.95–0.99) without significant evidence of nonlinearity (*p* = 0.30) (Figure 3B). For blood levels of AA, dihomo-γ-linolenic acid (DGLA), and γ-linolenic acid (GLA), no significant associations were observed.

## 4. Discussion

In the present study, we conducted a meta-analysis of prospective studies on the association between intake or blood levels of n-6 PUFAs and the risk of cancer. Our findings indicate that high blood n-6 PUFA levels are associated with a lower risk of cancer. Compared to people with the lowest blood levels of n-6 PUFAs, those with the highest blood levels of n-6 PUFAs had an 8% lower risk of cancer from any site. In the dose-response analysis, a 2% decreased risk of cancer was observed for a 5% increase in blood levels of n-6 PUFAs. When considering the site of cancer, breast cancer risk showed the strongest inverse association with blood levels of n-6 PUFAs. By type of n-6 PUFAs, the strongest inverse association was observed between blood levels of LA and risk of cancer. For sex, the inverse association was slightly stronger in women than in men. Regarding n-6 PUFA intake, no significant association was found in relation to the risk of cancer.

A previous meta-analysis published in 1998 summarized the results from studies on LA intake and breast, colorectal, and prostate cancer [6]. This study observed no significant association and concluded that high intake of LA did not substantially increase the risk of breast, colorectal, or prostate cancer in humans. Another meta-analysis investigating the association of intake and serum levels of LA with the risk of breast cancer suggested that LA may be associated with a decreased risk of breast cancer, reporting a nonsignificant inverse association [92]. We also found a null association between n-6 PUFA intake and the risk of cancer in the current meta-analysis.

Most studies included in our meta-analysis of n-6 PUFA intake assessed dietary intake using food frequency questionnaires. The few studies using a food diary, food record, or 24-h dietary recall showed a nonsignificant inverse [22] or positive [48,73] association between n-6 PUFA intake and the risk of cancer. Several previous studies investigating the association between fat intake and risk of cancer reported differences in results by dietary assessment methods. They found a significant association between fat intake and breast cancer risk when using food diaries or records, but a null association was observed when using food frequency questionnaires [8,93]. Dietary measurement error, which often occurs with food frequency questionnaires, might affect the results. Furthermore, fatty acids are particularly vulnerable to measurement errors because types of fat vary tremendously and assorted foods have different compositions of fatty acids [94]. Our results indicating a null association between n-6 PUFA intake and the risk of cancer may also have been affected by measurement errors. Because of the difficulties in estimating fat intake with dietary assessment, the use of circulating fatty acids can be an alternative approach for evaluating the association between fatty acids and the risk of disease. Although blood concentrations reflect absorption and metabolism as well as intake, PUFAs in blood may reflect intake relatively well because PUFAs were nearly derived from exogenous sources [32]. We found a significant inverse association between blood levels of n-6 PUFAs and risk of cancer, but a relatively small number of studies were included, compared with those assessing n-6 PUFA intake. Future studies on n-6 PUFAs and risk of cancer should consider blood levels of n-6 PUFAs as well as n-6 PUFA intake as an exposure.

The results from the subgroup analysis by cancer site showed that the inverse association between blood levels of n-6 PUFAs and the risk of breast cancer was stronger than that of other sites. Breast cancer is a common cancer that has been studied a lot in relation to fat intake, and thus the largest number of studies were included in our meta-analysis. A large prospective study involving 2982 breast cancer cases found a 19% lower risk of breast cancer in women with the highest blood levels of total n-6 PUFAs, compared to those with the lowest blood levels of total n-6 PUFAs [27]. When we analyzed by the type of n-6 PUFAs, LA showed a stronger inverse association with the risk of cancer than the other types of n-6 PUFAs. The differences in the risk of disease by type of n-6 PUFAs were also shown in previous studies. A cohort study including 2792 older adults found that high circulating LA was related to lower total and coronary heart disease (CHD) mortality, but no significant association was observed for other types of n-6 PUFAs [95]. LA accounts for the largest part of dietary intake or blood levels of n-6 PUFAs, and other n-6 PUFAs are produced from LA via desaturase and elongase [1]. In the subgroup analysis by sex, we found a stronger inverse association in women than in men, but this difference seems to be due to discrepancies in cancer sites because all breast cancer results were included in the subgroup analysis of women.

Although the evidence for the association between n-6 PUFAs and risk of disease was relatively limited compared to that of n-3 PUFAs, one meta-analysis including 310,602 individuals reported a low risk of CHD among people with high intake of LA [96]. Another meta-analysis including 22 observational studies found that LA biomarkers (blood/tissue) were inversely associated with the risk of nonfatal CHD [97]. Increasing numbers of studies have also suggested that n-6 PUFAs may be involved in anticancer processes [1]. Previous experimental studies observed that LA inhibited the proliferation and growth of colon cancer cells by enhancing cellular oxidant status and inducing mitochondrial dysfunction [98,99,100]. GLA was also observed to induce cancer cell apoptosis by altering mitochondrial metabolism and decreasing hexokinase and carnitine palmitoyltransferase I activities [101,102]. In addition, studies on cancer cells found that DGLA free radical derivatives from cyclooxygenase-catalyzed lipid peroxidation can inhibit cell growth and induce cell cycle arrest and apoptosis in human colon cancer cells [103]. Diets rich in n-6 PUFAs have been known to raise the risk of cancer, and it has been turned out to be mostly due to AA, which can produce eicosanoids with inflammatory properties [104]. Prostaglandin E_2_ (PGE_2_), which is derived from AA, has been found to increase tumor growth in previous studies [7]. Concerns about diets high in n-6 PUFAs were based on the hypothesis that LA, which takes up the largest portion of n-6 PUFA intake, is converted to AA [105]. However, the production of AA from LA is tightly regulated [106], and the extent of the conversion of LA to AA was observed to be as low as approximately 0.2% in tracer studies [107].

To the best of our knowledge, this is the first meta-analysis investigating the association between intakes or blood levels of n-6 PUFAs and risk of cancer from any site. As few studies have focused on n-6 PUFAs among the studies examining the association between fatty acids and the risk of cancer, our meta-analysis may provide useful information for future studies investigating the effect of n-6 PUFAs on the development of cancer. We tried to avoid recall bias by including only prospective studies in the meta-analysis. The inverse association between blood levels of n-6 PUFAs and risk of cancer was observed both in categorical (highest *versus* lowest) and dose-response meta-analyses. There was no significant heterogeneity, and the robustness of the results was identified through sensitivity analysis. In addition, there was no publication bias, which often could be of concern in meta-analysis. Despite these strengths, several limitations of this study should be noted. First, our meta-analysis of the association between n-6 PUFAs and the risk of cancer did not completely cover all cancer sites since previous studies have mostly focused on the risk of common cancers, such as breast, colorectal, or prostate cancer. Second, meta-analysis cannot solve problems of confounding that were inherent in the original studies. Although all studies included in the meta-analysis adjusted for age and most of the studies additionally controlled for major confounders such as BMI, smoking or alcohol, unknown or unmeasured factors might be a cause of confounding. Third, blood levels of n-6 PUFAs were measured at only at baseline, and thus, there is some possibility of change in blood levels of n-6 PUFAs during follow-up times. However, a previous study showed that a single measurement of blood levels of fatty acids reasonably reflected the blood levels over long periods [108].

## 5. Conclusions

In conclusion, the current meta-analysis indicated that individuals with high levels of n-6 PUFAs in blood (35.9% of total fatty acids, median) had a lower risk of cancer, compared to those with low levels of n-6 PUFAs in blood (28.7% of total fatty acids, median). The observed inverse association between blood levels of n-6 PUFAs and cancer risk was strong for breast cancer, LA, and women. We found no significant association between n-6 PUFA intake and the risk of cancer. Taken together our findings, n-6 PUFAs are unlikely to have an adverse effect on the development of cancer. Although it is not desirable to encourage people to eat high n-6 PUFAs, it might not be necessary to reduce n-6 PUFA intake excessively. Further well-designed prospective studies that use food records as a dietary assessment tool, consider blood levels of n-6 PUFAs as an exposure, or cover the risk of rare cancers are warranted to verify the effect of n-6 PUFAs on the risk of cancer.

## Figures and Tables

**Table 1:** Summary of pooled relative risks (RR) of cancer risk for n-6 polyunsaturated fatty acid (PUFA) intake.

Variable	No. of Studies	RR	95% CI	Heterogeneity	
				*I*^2^ (%)	*p*
**Cancer site**					
Any	47	1.02	0.99–1.05	38.9	0.01
Breast	13	1.00	1.00–1.01	0.0	0.49
Colorectal	11	0.99	0.90–1.09	36.0	0.11
Prostate	10	1.02	0.99–1.06	0.0	0.68
Pancreatic	4	0.99	0.86–1.14	0.0	0.80
Gynecological	4	1.04	0.90–1.19	0.0	0.81
Skin	3	1.02	0.80–1.29	79.7	0.03
Others	3	1.10	0.63–1.92	89.6	0.002
**Type of fatty acids**					
LA	30	0.99	0.94–1.04	23.2	0.13
AA	22	1.02	0.98–1.06	39.0	0.04
DGLA	4	1.10	0.93–1.29	0.0	0.93
GLA	1	0.92	0.53–1.60	-	-
**Sex**					
Men	17	1.03	0.98–1.08	28.1	0.14
Women	26	1.01	0.97–1.06	35.0	0.04

Abbreviations: LA, linoleic acid, AA, arachidonic acid; DGLA, dihomo-γ-linolenic acid; GLA, γ-linolenic acid.

**Table 2:** Summary of pooled relative risks (RR) of cancer risk for blood levels of n-6 PUFAs.

Variable	No. of Studies	RR	95% CI	Heterogeneity	
				*I*^2^ (%)	*p*
**Cancer site**					
Any	29	0.92	0.86–0.98	13.8	0.26
Breast	11	0.87	0.77–0.98	10.2	0.35
Prostate	10	0.94	0.84–1.05	39.3	0.10
Colorectal	4	0.92	0.77–1.10	0.0	0.55
Others	4	0.90	0.75–1.08	0.0	0.47
**Type of fatty acids**					
LA	28	0.91	0.82–1.00	42.2	0.01
AA	17	0.98	0.91–1.05	0.0	0.66
DGLA	26	0.99	0.88–1.12	30.5	0.11
GLA	17	0.94	0.83–1.06	23.4	0.19
**Sex**					
Men	13	0.92	0.83–1.02	30.1	0.14
Women	13	0.88	0.79–0.97	0.0	0.48

Abbreviations: PUFA, polyunsaturated fatty acid; LA, linoleic acid, AA, arachidonic acid; DGLA, dihomo-γ-linolenic acid; GLA, γ-linolenic acid.

## Supplementary Materials

The following are available online at https://www.mdpi.com/2072-6643/12/9/2523/s1, Table S1: Characteristics of Prospective Studies Included in the Meta-Analysis of n-6 polyunsaturated fatty acid (PUFA) intake and colorectal cancer, Table S2: Characteristics of Prospective Studies Included in the Meta-Analysis of blood levels of n-6 polyunsaturated fatty acids (PUFAs) and colorectal cancer Figure S1: Flow chart of study selection
