# Supplementary material for: N-6 Polyunsaturated Fatty Acids and Risk of Cancer: Accumulating Evidence from Prospective Studies
Source: Nutrients. 2020 Aug 20;12(9):2523. doi: 10.3390/nu12092523 (PMC7551408; doi:10.3390/nu12092523)
Supplement: Supplementary file 1 [file nutrients-12-02523-s001.pdf]

Supplementary Table 1. Characteristics of Prospective Studies Included in the Meta-Analysis of n-6 polyunsaturated fatty acid (PUFA) intake and colorectal caner

First author, year	Country	Cohort name	Follow-up period	Age at baseline (years)	Study size		Cancer site	N-6 PUFAs	Adjustment for covariates
					Subjects	No. of cases			
Holmes, 1999 (1)	US	Nurses' Health Study (NHS)	14 years	30-55	77,519	2,097	Breast	LA<br>AA	Age, energy, energy-adjusted vitamin A intake, alcohol intake, time period, height, parity, age at first birth, weight change since age 18 years, age at menopause, menopausal status and use of hormone replacement therapy, family history, benign breast disease, age at menarche.
Sieri, 2002 (2)	Italy	ORDET study	5.5 years	41–70	3,367	56	Breast	LA	Age, daylight-saving period at recruitment, recruitment center, recruitment date, parity, place of birth, and level of education, total fat, BMI, waist-to-hip ratio
Voorrips, 2002 (3)	Netherlands	Netherlands Cohort Study	6.3 years	55–69	2,539	941	Breast	LA<br>AA	Age, history of benign breast disease, maternal breast cancer, breast cancer in one or more sisters, age at menarche, age at menopause, oral contraceptive use, parity, age at first childbirth, Quetelet index, education, alcohol use, current cigarette smoking, total energy intake, and total energy adjusted fat intake.
Wirfält, 2002 (4)	Sweden	Malmo¨ Diet and Cancer	1991-1999	≥50	910	237	Breast	N-6 PUFAs	Age, year and month of data collection, quintiles of n-6, n-3, monounsaturated and saturated fatty acids, total energy, past food habit change, BMI, height, waist circumference, age at birth of first child, current hormone therapy, alcohol habits, and educational status.
Gago-Dominguez, 2003 (5)	Singapore	Singapore Chinese Health Study	5.3 years	45–74	35,298	314	Breast	N-6 PUFAs	Age at baseline interview, year of recruitment, dialect group, education, daily alcohol drinker, family history of breast cancer, age when period became regular, and number of live births

Wakai, 2005 (6)	Japan	Japan Collaborative Cohort	7.6 years	40-79	26,291	129	Breast	N-6 PUFAs	Age, study area, educational level, family history of breast cancer, age at menarche, age at menopause, age at first birth, parity, use of exogenous female hormones, alcohol consumption, smoking, consumption of green leafy vegetables, daily walking, height, BMI, and total energy intake
Thiébaut, 2009, a (7)	France	E3N	8 years	40-65	56,007	1,650	Breast	N-6 PUFAs<br>LA<br>AA	Age, nonalcohol energy and ethanol intakes, smoking history, history of benign breast disease, history of breast cancer in first-degree relatives, age at menarche, parity, BMI, menopausal status, age at menopause and use of menopausal hormone treatment
Murff, 2011 (8)	China	Shanghai Women's Health Study (SWHS)	1996-2007	40-70	72,571	712	Breast	LA<br>AA	Age, BMI, total energy, family history of breast cancer, alcohol use, tobacco use, education, use of hormone replacement therapy, personal history of diabetes, menopausal status, age at menopause, age at menarche, parity, age at first pregnancy, level of physical activity, red meat intake, fish intake and vitamin E intake
Park, 2012 (9)	US	Multiethnic Cohort Study (MEC)	12 years	45-75	85,089	3,885	Breast	N-6 PUFAs<br>LA<br>AA	Age at cohort entry, time on study, ethnicity, family history of breast cancer, education, BMI, age at menarche, age at first live birth, number of children, age at and type of menopause, hormone replacement therapy, smoking status, energy intake, and alcohol use.
Sczaniecka, 2012 (10)	US	VITamins And Lifestyle (VITAL) Cohort	6 years	50-76	30,252	772	Breast	LA<br>AA	Age, race, education, height, body mass index, age at menarche, age at first birth, age at menopause, history of hysterectomy, years of combined hormone therapy, years of estrogen hormone therapy, family history of breast cancer, mammography, history of benign breast biopsy, regular use of nonsteroidal antiinflammatory drugs, exercise, alcohol consumption, vegetable intake, fruit intake, and total energy.

Kiyabu, 2015 (11)	Japan	Japan Public Health Center-based prospective study (JPHC)	14.1 years	45–74	38,234	556	Breast	N-6 PUFAs	Age, area, BMI, age at menarche, age at first birth, parity, menopausal age, menopausal status at baseline, use of exogenous female hormones which include oral contraceptives and menopausal hormone treatment, leisure-time physical activity, smoking status, alcohol intake and total energy-adjusted intake of isoflavones
Bassett, 2016 (12)	Australia	Melbourne Collaborative Cohort Study	8.9 years	27-80	2,491	470	Breast	N-6 PUFAs<br>LA<br>DGLA<br>AA	Age, country of birth, menopausal status, age at menarche, parity and lactation, oral contraceptive use, hormone therapy use, physical activity, alcohol consumption, smoking status, education, family history of cancer, total energy intake from food and stratified by BMI category
Sellem, 2019 (13)	France	NutriNet-Santé study	2009-2017	≥ 45	44,039	545	Breast<br>Prostate<br>All	N-6 PUFAs	Age, sex, BMI, height, physical activity, smoking status, number of 24-h dietary records, alcohol intake, energy intake without alcohol, family history of cancer, educational level, total lipid intake, fruit and vegetable intake
Weijenberg, 2007 (14)	Netherlands	Netherlands Cohort Study (NLCS)	7.3 years	55-69	3,500	434	Colorectal	LA	Age, sex, BMI, smoking, energy intake, family history of colorectal cancer
Butler, 2009 (15)	Singapore	Singapore Chinese Health Study	9.8 years	45–74	61,321	961	Colorectal	N-6 PUFAs	Age at interview, sex, dialect group, interview year, diabetes at baseline, smoking history, BMI, alcohol intake, education, any weekly physical activity, first degree relative diagnosed with colorectal cancer, and total daily energy intake
Murff, 2009 (16)	China	Shanghai Women's Health Study (SWHS)	1996-2007	40-70	73,242	396	Colorectal	N-6 PUFAs<br>LA<br>AA	Age, energy intake, total energy-adjusted n-6 PUFA intake, energy-adjusted ratio of total n-6 PUFA to n-3 PUFA intake, BMI, current smoker, alcohol use, regular physical activity in past 5 y, total energy-adjusted red meat intake, menopausal status, hormone replacement therapy use, multivitamin use, and aspirin use

Daniel, 2009 (17)	US	Cancer Prevention Study-II Nutrition Cohort	6 years	70 M<br>68 F, mean	99,080	869	Colorectal	N-6 PUFAs	Age, energy, recreational physical activity, NSAID use, colorectal screening, BMI, and red and processed meat, low-fat dairy, fruit, and vegetable intake
Sasazuki, 2011 (18)	Japan	The Japan Public Health Center (JPHC)	9.3 years	40–69	98,466	1,268	Colorectal	N-6 PUFAs	Age, area, BMI, smoking status, alcohol drinking, past history of or medication use for diabetes mellitus, METs, screening for colorectal cancer, total calorie, intake of calcium, vitamin D, fiber and red meat.
Key, 2012 (19)	UK	UK Dietary Cohort Consortium	1985-2006	61.7, mean	2,415	547	Colorectal	N-6 PUFAs	Age, date of diary, sex, height, weight, energy intake, alcohol intake, fiber intake, smoking, education, social class, physical activity
Song, 2014, NHS (20)	US	Nurses' Health Study (NHS)	24 years	30–55	76,386	1,469	Colorectal	N-6 PUFAs	Age, calendar year, family history of colorectal cancer, prior lower gastrointestinal endoscopy, pack-years of smoking before age 30, BMI, physical activity, current multivitamin use, postmenopausal status and hormone use, regular aspirin or NSAID use, total caloric intake, red meat, process meat, alcohol consumption, energy-adjusted intake of folate, calcium, vitamin D and total fiber.
Song, 2014, HPFS (20)	US	Health Professionals Follow-up Study (HPFS)	26 years	40–75	47,143	987	Colorectal	N-6 PUFAs	Age, calendar year, family history of colorectal cancer, prior lower gastrointestinal endoscopy, pack-years of smoking before age 30, BMI, physical activity, current multivitamin use, regular aspirin or NSAID use, total caloric intake, red meat, process meat, alcohol consumption and energy-adjusted intake of folate, calcium, vitamin D and total fiber
Hodge, 2015 (21)	Australia	Melbourne Collaborative Cohort Study (MCCS)	9 years	40–69	41,514	395	Colorectal	N-6 PUFAs<br>LA<br>DGLA<br>AA	Age, education, alcohol intake, smoking status, physical activity, total energy intake and stratified by: sex, ethnicity (Southern-European migrant vs. not) and family history of cancer

Kraja, 2015 (22)	Netherlands	Rotterdam Study	14.6 years	≥55	4,967	222	Colorectal	N-6 PUFAs	Age, sex, energy-adjusted dietary fiber intake, intake of vegetable, trans fat, fruit, alcohol, and sodium
Navarro, 2016 (23)	US	Women's Health Initiative prospective cohort (WHI)	11.7 years	50–79	134,017	1,952	Colorectal	N-6 PUFAs	Age, total energy intake, BMI, education, family history of colorectal cancer, history of colonoscopy, current NSAID use, alcohol intake, smoking history, physical activity, ever use of hormone therapy, folate, calcium, and red meat intake, study component, randomization assignment and treatment arm.
Shin, 2020 (24)	Sweden	Swedish Women's Lifestyle and Health cohort (WLH)	21.3 years	29-49	48,233	344	Colorectal	N-6 PUFAs<br>LA<br>AA	Age, BMI, education, smoking history, alcohol intake
Schuurman, 1999 (25)	Netherlands	Netherlands Cohort Study	6.3 years	55–69	58,279	642	Prostate	LA<br>AA	Age, family history of prostate carcinoma, socioeconomic status, total energy intake, and total energy-adjusted fat intake.
Männistö, 2003 (26)	Finland	Alpha-Tocopherol, Beta-Carotene Cancer Prevention Study	6.1 years	50-69	29,133	246	Prostate	LA<br>GLA<br>DGLA<br>AA	Age, trial supplementation group, area of residence, education, BMI, alcohol consumption, and the number of years of smoking.
Laaksonen, 2004 (27)	Finland	Kuopio Ischaemic Heart Disease Risk Factor Study	12.6 years	42-60	2,002	46	Prostate<br>All	LA	Age, year of examination, BMI, fasting serum insulin and blood glucose levels, nonesterified fatty acid concentrations.

Leitzmann, 2004 (28)	US	Health Professionals Follow-Up Study	14 years	40–75	47,866	2,965	Prostate	LA<br>AA	Age, time period, major ancestry, family history of prostate cancer, BMI at age 21 y, height, history of type 2 diabetes, history of vasectomy, cigarette smoking in the previous decade, vigorous physical activity, intake of total energy, percentage of energy from protein intake, percentage of energy from monounsaturated fat intake, percentage of energy from saturated fat intake, percentage of energy from trans unsaturated fat intake, and intakes of calcium, supplemental vitamin E, and lycopene. other polyunsaturated fatty acids.
Neuhouser, 2007 (29)	US	Carotene and Retinol Efficacy Trial (CARET)	11 years	50-69	12,025	811	Prostate	N-6 PUFAs	Age, race/ethnicity, energy intake, BMI, smoking, family history
Park, 2007 (30)	US	Multiethnic Cohort Study (MEC)	8 years	≥45	82,483	4,404	Prostate	N-6 PUFAs	Age, time on study, ethnicity, family history of prostate cancer, education, BMI, smoking status and energy intake
Wallström, 2007 (31)	Sweden	Malmö Diet and Cancer cohort	11 years	45-73	10,564	817	Prostate	N-6 PUFAs<br>LA<br>AA	Age, diabetes, waist circumference, height, living alone/with partner/with other , educational level, alcohol habits, BMI, smoking history, birth country (Sweden/other; categorical), total calcium intake, consumption of fruits, vegetables, red meat
Bassett, 2013 (32)	Australia	Melbourne Collaborative Cohort	8.9 years	27-80	1,717	464	Prostate	N-6 PUFAs<br>LA<br>DGLA<br>AA	Age, country of birth, education, alcohol intake, physical activity, total energy intake from food and family history of cancer and stratified by smoking status.
Pelser, 2013 (33)	US	NIH-American Association of Retired Persons (AARP) Diet and Health Study	9 years	50-71	288,268	23,281	Prostate	N-6 PUFAs<br>LA<br>AA	Age at entry, race, family history of prostate cancer, education, marital status, prostate-specific antigen testing in the past 3 years, physical activity, smoking, self-reported diabetes, BMI at baseline, calories, alcohol, and intake of tomatoes.

Stolzenberg-Solomon, 2002 (34)	Finland	Alpha-Tocopherol, Beta-Carotene Cancer Prevention Study	10.2 years	50-69	27,111	163	Pancreatic	LA	Age, energy intake by the residual method, years of smoking, energy-adjusted saturated fat intake
Michaud, 2003 (35)	US	Nurses' Health Study	18 years	30-55	88,802	178	Pancreatic	LA	Age, pack-years of smoking, BMI, history of diabetes mellitus, caloric intake, height , physical activity, menopausal status, glycemic load intake
Heinen, 2009 (36)	Netherlands	Netherlands Cohort Study (NLCS)	13.3 years	55-69	120,852	350	Pancreatic	LA	Age, gender, energy, smoking, alcohol, history of diabetes mellitus, history of hypertension , BMI, vegetables, fruit
Thiébaut, 2009, b (37)	US	National Institutes of Health – AARP Diet and Health Study	6.3 years	50-71	308,736 men 216,737 women	865 men 472 women	Pancreatic	N-6 PUFAs LA AA	Age, sex, total energy intake, smoking history, BMI, self-reported history of diabetes
Jain, 2000 (38)	Canada	National Breast Screening Study	10.3 years	40-59	3,918	221	Endometrial	LA	Age, total energy, BMI, ever smoked, used oral contraceptives, used hormone replacement therapy, university education, live births, age at menarche
Brasky, 2015 (39)	US	Women's Health Initiative (WHI)	13 years	50-79	87,360	1,253	Endometrial	N-6 PUFAs LA AA	Age, total energy, clinical trial /observational study intervention assignment, US region, race, education, BMI, smoking, alcohol, physical activity, age at menarche, age at first birth, age at menopause, parity, duration of combined menopausal hormone therapy, duration of estrogen-alone hormone therapy, duration of oral contraceptive use, oophoerectomy status, family history of endometrial cancer, history of diabetes
Brasky, 2016 (40)	US	Black Women's Health Study (BWHS)	18 years	21-69	47,602	282	Endometrial	N-6 PUFAs LA AA	Age, time period, total energy intake, US region, education, BMI, physical activity, alcohol consumption, smoking, fruit consumption, vegetable consumption, age at menarche, age at menopause, parity, age at first birth, duration of combined hormone therapy, duration of estrogen-alone hormone therapy, duration of oral contraceptive use, diabetes

Bertone, 2002 (41)	US	Nurses' Health Study (NHS)	1980-1996	30–55	80,258	301	Ovarian	LA<br>AA	Age, parity, age at menarche, oral contraceptive use, duration, menopausal status/postmenopausal hormone use, tubal ligation, smoking status
Wallingford, 2012 (42)	Australia	Nambour Trial and Follow-Up Study	10 years	54, mean	1,322	325	Skin	N-6 PUFAs<br>LA<br>AA	Age, sex, freckling on back, elastosis of neck, treatment allocation
Park, 2018 (43)	US	Nurses' Health Study (NHS), Health Professionals Follow-up Study (HPFS)	18 years	30–55 (NHS)<br>40-75 (HPFS)	75,311 (NHS)<br>48,516 (HPFS)	794 (NHS)<br>736 (HPFS)	Skin	N-6 PUFAs	Age, family history of melanoma, natural hair color, number of arm moles, sunburn susceptibility as a child/adolescent, number of lifetime blistering sunburns, cumulative UV flux since baseline, BMI, physical activity, smoking status, incident SCC, incident BCC, personal history of non-skin cancer, intakes of total energy, alcohol, caffeine, and citrus fruits, menopausal status, postmenopausal hormone use, saturated, monounsaturated, and omega-3 fats, cholesterol
Luu, 2018 (44)	China	Shanghai Women's Health Study (SWHS), Shanghai Men's Health Study (SMHS)	11 years	40-70 (SWHS)<br>40-74 (SMHS)	121,970	1,496	Lung	N-6 PUFAs<br>LA<br>AA	Age, ever smoking status, ever drinking status, BMI, physical activity status, vitamin supplemental use, smoking packs-year (SMHS only), menopausal status and hormone replacement therapy (SWHS only)
Koh, 2016 (45)	China	Singapore Chinese Health Study	14 years	45–74	63,257	488	Liver	N-6 PUFAs	Age, sex, dialect, year of interview, educational level, BMI, smoking status, alcohol use, coffee drinking status, baseline history of self-reported diabetes, total energy, dietary protein, saturated fat, monounsaturated fat, omega-3 PUFA
Yang, 2020 (46)	US	Nurses' Health Study (NHS), Health Professionals Follow-up Study (HPFS)	26.6 years	62, mean	138,483	160	Liver	N-6 PUFAs	Age, sex, race, physical activity, BMI, smoking status, aspirin use, type 2 diabetes, alcohol intake, total coffee intake, total calorie intake

Abbreviations: PUFA, polyunsaturated fatty acid; LA, linoleic acid, AA, arachidonic acid; DGLA, dihomo-ɤ-linolenic acid; GLA, ɤ-linolenic acid.

Supplementary Table 2. Characteristics of Prospective Studies Included in the Meta-Analysis of blood levels of n-6 polyunsaturated fatty acids (PUFAs) and colorectal caner

First author, year	Country	Cohort name	Follow-up period	Age at baseline (years)	Study size		Cancer site	N-6 PUFAs	Adjustment for covariates
					No. of controls	No. of cases			
Chajés, 1999 (47)	Sweden	Västerbotten Intervention Project, Monitoring of Trends and Cardiovascular Disease study, Mammary-Screening Project	1986-1997	55, mean	388	196	Breast	N-6 PUFAs<br>AA	Age, duration of follow-up, age at menarche, age at first full-term pregnancy, number of children, use of hormone replacement therapy, height and weight.
Pala, 2001 (48)	Italy	ORDET study	5.5 years	42-69	141	71	Breast	N-6 PUFAs<br>LA<br>GLA<br>DGLA<br>AA	Age, menopausal status at recruitment, daylight-saving period at blood sampling, recruitment center, recruitment date, BMI, waist hip ratio, age at menarche, age at first childbirth, age at menopause, months of lactation, parity, and educational level
Saadatian-Elahi, 2002 (49)	US	New York University Women's Health Study	4.3 years	34-65	197	197	Breast	N-6 PUFAs<br>LA<br>GLA<br>DGLA<br>AA	Age at recruitment, menopausal status at baseline, date of baseline blood sampling, number of blood samplings before a case's date of diagnosis, age at first full-term birth, family history of breast cancer, history of benign breast disease, and total cholesterol.

Rissanen, 2003 (50)	Finland	Mobile Clinic Health Examination Survey	10 years	19-89	110	58	Breast	N-6 PUFAs<br>LA<br>GLA<br>DGLA	Age, menopausal status, municipality, BMI, serum cholesterol, smoking, alcohol consumption, number of pregnancies, parity, leisure-time exercise, and education
Wirfält, 2004 (51)	Sweden	Malmo¨ Diet and Cancer	1991-1999	≥50	673	237	Breast	LA<br>DGLA<br>AA	Age, screening date
Chajés, 2008 (52)	France	E3N	7 years	40-65	702	363	Breast	N-6 PUFAs<br>LA<br>GLA<br>AA	Age, menopausal status at blood collection, fasting status at blood collection, study center, date of blood collection, BMI, alcohol consumption, height, menopausal hormone use, educational level, parity, family history of breast cancer, and history of benign breast disease.
Takata, 2009 (53)	US	β-Carotene and Retinol Efficacy Trial (CARET)	1985-1997	50-69	257	130	Breast	N-6 PUFAs<br>LA<br>GLA<br>AA	Age, study center, year of the enrollment, smoking status at baseline and blood draw, BMI, overweight, obese, intervention arm, and alcohol use at the time of blood draw
Pouchieu, 2014 (54)	France	Supplementation en Vitamines et Mine´raux Antioxydants study (SU.VI.MAX)	3.7 years	51, mean	250	250	Breast<br>All	N-6 PUFAs<br>LA<br>GLA<br>DGLA<br>AA	Age, sex, number of dietary records, intervention group of the trial, number of dietary records, BMI, height, smoking status, physical activity, alcohol intake, educational level, family history of breast cancer, menopausal status, use of hormonal treatment for menopause and number of children

Bassett, 2016 (12)	Australia	Melbourne Collaborative Cohort Study	8.9 years	27-80	2,491 subjects	470	Breast	N-6 PUFAs<br>LA<br>DGLA<br>AA	Age, country of birth, menopausal status, age at menarche, parity and lactation, oral contraceptive use, hormone therapy use, physical activity, alcohol consumption, smoking status, education, family history of cancer, total energy intake from food and stratified by BMI category
Chajés, 2017 (55)	Europe	European Prospective Investigation into Cancer and Nutrition study (EPIC)	11.5 years	54, mean	2,982	2,982	Breast	N-6 PUFAs<br>LA<br>GLA<br>AA	Age, center, menopausal status, time of the day at blood collection, fasting status, phase of the menstrual cycle, date at blood collection, years of education, BMI, height, menopausal hormone use at baseline, alcohol at baseline, age at first birth and parity combined, energy intake, family history of breast cancer.
Hirko, 2018 (56)	US	Nurses' Health Study II	1996-2007	32-54	794	794	Breast	N-6 PUFAs<br>LA<br>GLA<br>DGLA<br>AA	Age, menopausal status at blood draw and diagnosis, self-reported race/ ethnicity, fasting status, and month, time of day of blood collection, luteal day, menopausal hormone therapy use at blood draw. age at menarche, age at first birth /parity, breastfeeding, family history of breast cancer, history of biopsy-confirmed benign breast disease, BMI at age 18, weight change between age 18 and blood collection, alcohol consumption, physical activity

Kojima, 2005 (57)	Japan	Japan Collaborative Cohort Study	7.1 years	40–79	481	169	Colorectal	N-6 PUFAs<br>LA<br>GLA<br>DGLA<br>AA	Age, participating institution, family history of colorectal cancer in first-degree relatives, BMI, education, smoking and alcohol drinking history, green leafy vegetable intake, and physical exercise.
Hall, 2007 (58)	US	Physicians' Health Study (PHS)	1982-1995	40-84	282	178	Colorectal	N-6 PUFAs	Age, smoking status, BMI, multivitamin use, history of diabetes, random assignment to aspirin or placebo, vigorous exercise, alcohol intake, and quartile of red meat intake and long-chain n-3 PUFAs.
Hodge, 2015 (21)	Australia	Melbourne Collaborative Cohort Study (MCCS)	9 years	40–69	4,205	395	Colorectal	N-6 PUFAs	Age, education, alcohol intake, smoking status, physical activity, total energy intake and stratified by: sex, ethnicity (Southern-European migrant vs. not) and family history of cancer
Butler, 2017 (59)	Singapore	Singapore Chinese Health Study	3.3 years	45–74	350	350	Colorectal	LA<br>GLA<br>DGLA<br>AA	Age, sex, dialect group, date of baseline interview, and date of biospecimen collection, BMI, smoking, education level, alcohol use, weekly physical activity , history of diabetes , and use of NSAIDs.
Harvei, 1997 (60)	Norway		11.6 years	50, mean	282	141	Prostate	N-6 PUFAs<br>LA<br>DGLA<br>AA	Age, place of residence, date of blood sample,

Männistö, 2003 (26)	US	Alpha-Tocopherol, Beta-Carotene Cancer Prevention Study	6.1 years	50-69	198	198	Prostate	LA<br>GLA<br>DGLA<br>AA	Age, trial supplementation group, area of residence, education, BMI, alcohol consumption, and the number of years of smoking.
Laaksonen, 2004 (27)	Finland	Kuopio Ischaemic Heart Disease Risk Factor Study	12.6 years	42-60	2,002	151	Prostate<br>All	N-6 PUFAs<br>LA	Age, year of examination, BMI, fasting serum insulin and blood glucose levels, nonesterified fatty acid concentrations
Chavarro, 2007 (61)	US	Physician's Health Study (PHS)	13 years	40-84	476	476	Prostate	LA<br>GLA<br>DGLA<br>AA	Age, smoking status, length of follow-up
Crowe, 2008 (62)	Europe	European Prospective Investigation into Cancer and Nutrition (EPIC)	4.2 years	60, mean	962	962	Prostate	LA<br>DGLA<br>AA	Age, study center, time of day of blood collection, time between blood draw and last consumption of food or drink, BMI, smoking, alcohol intake, education, marital status, and physical activity
Park, 2009 (63)	US	Multiethnic Cohort (MEC)	1.9 years	45-75	729	376	Prostate	N-6 PUFAs<br>LA<br>AA	Age, geographic location (Hawaii or California), race/ethnicity, birth year, date of blood draw, time of blood draw, fasting hours, family history of prostate cancer, BMI, education
Brasky, 2011 (64)	US	Prostate Cancer Prevention Trial	7 years	55–84	1,803	1,658	Prostate	N-6 PUFAs<br>LA<br>AA	Age, treatment, prostate cancer family history, race, diabetes, BMI, alcohol, and treatment arm

Bassett, 2013 (32)	Australia	Melbourne Collaborative Cohort	8.9 years	27-80	1,717 subjects	464	Prostate	N-6 PUFAs<br>LA<br>DGLA<br>AA	Age, country of birth, education, alcohol intake, physical activity, total energy intake from food and family history of cancer and stratified by smoking status.
Brasky, 2013 (65)	US	Selenium and Vitamin E Cancer Prevention Trial (SELECT)	2001-2011	≥50	1,393	834	Prostate	LA<br>AA	Age, race, education, history of diabetes, family history of prostate cancer, and SELECT intervention arm
Cheng, 2013 (66)	US	Carotene and Retinol Efficacy Trial (CARET)	7 years	45-69	1,398	641	Prostate	N-6 PUFAs<br>LA<br>GLA<br>DGLA<br>AA	Age, race/ethnicity, CARET randomization assignment, family history of prostate cancer in first-degree relatives, alcohol consumption, smoking status, smoking pack-years, BMI
Matejcic, 2018 (67)	Europe	European Prospective Investigation into Cancer and Nutrition (EPIC)	11.7 years	35-70	375	375	Pancreatic	N-6 PUFAs<br>LA<br>GLA<br>DGLA<br>AA	Age at blood collection, study center, sex, date and time at blood collection, length of follow-up, fasting status, use of pill/hormonal replacement treatment, BMI, height, history of diabetes mellitus, smoking status, alcohol intake, education, physical activity.
Wallingford, 2013 (68)	Australia	Nambour Skin Cancer Study	11 years	54, mean	1,191 subjects	337	Skin	N-6 PUFAs<br>LA<br>AA	Age, sex, elastosis of neck, total solar keratoses, treatment allocation
Morimoto, 2012 (69)	US	Multiethnic Cohort Study (MEC)	3.1 years	45-75	549	275	Lymphoma	N-6 PUFAs<br>LA<br>AA	Age, sex, ethnicity, location, date of blood draw, time of day of blood draw, fasting hours prior to blood draw
Chajés, 2011 (70)	Europe	European Prospective Investigation into	1992-2002	59, mean	326	238	Gastric	N-6 PUFAs<br>LA<br>DGLA<br>AA	Age, sex, study center, and date of blood donation, Helicobacter pylori infection status, BMI, status, duration, and intensity of

	Cancer and Nutrition (EPIC)		smoking, physical activity, level of schooling, energy intake

Abbreviations: PUFA, polyunsaturated fatty acid; LA, linoleic acid, AA, arachidonic acid; DGLA, dihomo-γ-linolenic acid; GLA, γ-linolenic acid.

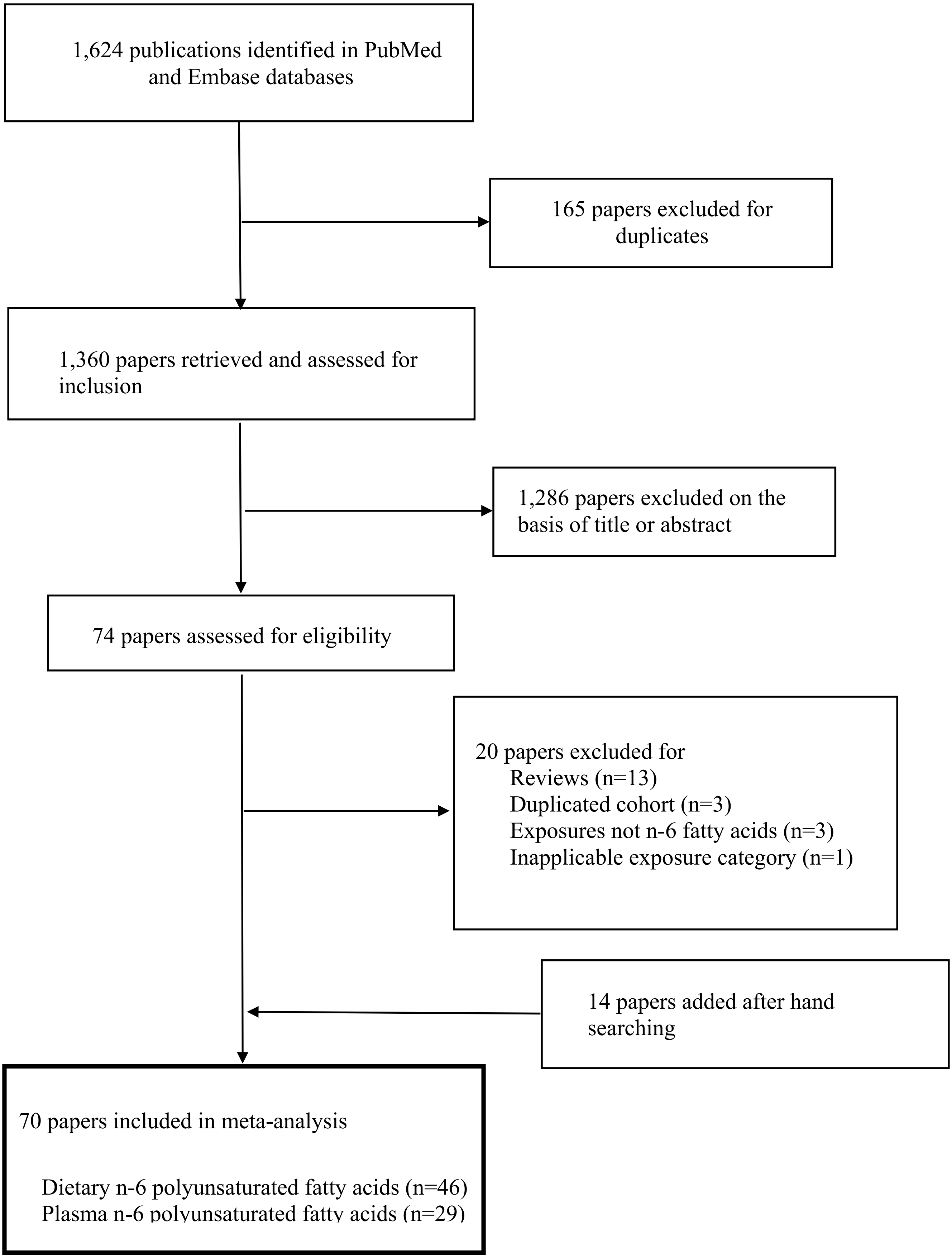

Supplementary Figure 1. Flow chart of study selection

References

1. Holmes MD, Hunter DJ, Colditz GA, Stampfer MJ, Hankinson SE, Speizer FE, *et al.* Association of dietary intake of fat and fatty acids with risk of breast cancer. JAMA **1999**;281(10):914-20 doi 10.1001/jama.281.10.914.
2. Sieri S, Krogh V, Muti P, Micheli A, Pala V, Crosignani P, *et al.* Fat and protein intake and subsequent breast cancer risk in postmenopausal women. Nutr Cancer **2002**;42(1):10-7 doi 10.1207/s15327914nc421_2.
3. Voorrips LE, Brants HA, Kardinaal AF, Hiddink GJ, van den Brandt PA, Goldbohm RA. Intake of conjugated linoleic acid, fat, and other fatty acids in relation to postmenopausal breast cancer: the Netherlands Cohort Study on Diet and Cancer. Am J Clin Nutr **2002**;76(4):873-82 doi 10.1093/ajcn/76.4.873.
4. Wirfalt E, Mattisson I, Gullberg B, Johansson U, Olsson H, Berglund G. Postmenopausal breast cancer is associated with high intakes of omega6 fatty acids (Sweden). Cancer Causes Control **2002**;13(10):883-93.
5. Gago-Dominguez M, Yuan JM, Sun CL, Lee HP, Yu MC. Opposing effects of dietary n-3 and n-6 fatty acids on mammary carcinogenesis: The Singapore Chinese Health Study. Br J Cancer **2003**;89(9):1686-92 doi 10.1038/sj.bjc.6601340.
6. Wakai K, Tamakoshi K, Date C, Fukui M, Suzuki S, Lin Y, *et al.* Dietary intakes of fat and fatty acids and risk of breast cancer: a prospective study in Japan. Cancer Sci **2005**;96(9):590-9 doi 10.1111/j.1349-7006.2005.00084.x.
7. Thiebaut AC, Chajes V, Gerber M, Boutron-Ruault MC, Joulin V, Lenoir G, *et al.* Dietary intakes of omega-6 and omega-3 polyunsaturated fatty acids and the risk of breast cancer. Int J Cancer **2009**;124(4):924-31 doi 10.1002/ijc.23980.
8. Murff HJ, Shu XO, Li H, Yang G, Wu X, Cai H, *et al.* Dietary polyunsaturated fatty acids and breast cancer risk in Chinese women: a prospective cohort study. Int J Cancer **2011**;128(6):1434-41 doi 10.1002/ijc.25703.
9. Park SY, Kolonel LN, Henderson BE, Wilkens LR. Dietary fat and breast cancer in postmenopausal women according to ethnicity and hormone receptor status: the Multiethnic Cohort Study. Cancer Prev Res (Phila) **2012**;5(2):216-28 doi 10.1158/1940-6207.CAPR-11-0260.
10. Sczaniecka AK, Brasky TM, Lampe JW, Patterson RE, White E. Dietary intake of specific fatty acids and breast cancer risk among postmenopausal women in the VITAL cohort. Nutr Cancer **2012**;64(8):1131-42 doi 10.1080/01635581.2012.718033.
11. Kiyabu GY, Inoue M, Saito E, Abe SK, Sawada N, Ishihara J, *et al.* Fish, n - 3 polyunsaturated fatty acids and n - 6 polyunsaturated fatty acids intake and breast cancer risk: The Japan Public Health Center-based prospective study. Int J Cancer **2015**;137(12):2915-26 doi 10.1002/ijc.29672.
12. Bassett JK, Hodge AM, English DR, MacInnis RJ, Giles GG. Plasma phospholipids fatty acids, dietary fatty acids, and breast cancer risk. Cancer Causes Control **2016**;27(6):759-73 doi 10.1007/s10552-016-0753-2.
13. Sellem L, Srour B, Guéraud F, Pierre F, Kesse-Guyot E, Fiolet T, *et al.* Saturated, mono- and polyunsaturated fatty acid intake and cancer risk: results from the French prospective cohort NutriNet-Santé. European Journal of Nutrition **2019**;58(4):1515-27 doi 10.1007/s00394-018-1682-5.
14. Weijenberg MP, Luchtenborg M, de Goeij AF, Brink M, van Muijen GN, de Bruine AP, *et al.* Dietary fat and risk of colon and rectal cancer with aberrant MLH1 expression, APC or KRAS genes. Cancer Causes Control **2007**;18(8):865-79 doi 10.1007/s10552-007-9032-6.
15. Butler LM, Wang R, Koh WP, Stern MC, Yuan JM, Yu MC. Marine n-3 and saturated fatty acids in relation to risk of colorectal cancer in Singapore Chinese: a prospective study. Int J Cancer **2009**;124(3):678-86 doi 10.1002/ijc.23950.
16. Murff HJ, Shu XO, Li H, Dai Q, Kallianpur A, Yang G, *et al.* A prospective study of dietary polyunsaturated fatty acids and colorectal cancer risk in Chinese women. Cancer Epidemiol Biomarkers Prev **2009**;18(8):2283-91 doi 10.1158/1055-9965.epi-08-1196.
17. Daniel CR, McCullough ML, Patel RC, Jacobs EJ, Flanders WD, Thun MJ, *et al.* Dietary intake of omega-6 and omega-3 fatty acids and risk of colorectal cancer in a prospective cohort of U.S. men and women. Cancer Epidemiol Biomarkers Prev **2009**;18(2):516-25 doi 10.1158/1055-9965.epi-08-0750.
18. Sasazuki S, Inoue M, Iwasaki M, Sawada N, Shimazu T, Yamaji T, *et al.* Intake of n-3 and n-6 polyunsaturated fatty acids and development of colorectal cancer by subsite: Japan Public Health Center-based prospective study. Int J Cancer **2011**;129(7):1718-29 doi 10.1002/ijc.25802.
19. Key TJ, Appleby PN, Masset G, Brunner EJ, Cade JE, Greenwood DC, *et al.* Vitamins, minerals, essential fatty acids and colorectal cancer risk in the United Kingdom Dietary Cohort Consortium. Int J Cancer **2012**;131(3):E320-5 doi 10.1002/ijc.27386.
20. Song M, Chan AT, Fuchs CS, Ogino S, Hu FB, Mozaffarian D, *et al.* Dietary intake of fish, omega-3 and omega-6 fatty acids and risk of colorectal cancer: A prospective study in U.S. men and women. Int J Cancer **2014**;135(10):2413-23 doi 10.1002/ijc.28878.
21. Hodge AM, Williamson EJ, Bassett JK, MacInnis RJ, Giles GG, English DR. Dietary and biomarker estimates of fatty acids and risk of colorectal cancer. Int J Cancer **2015**;137(5):1224-34 doi 10.1002/ijc.29479.
22. Kraja B, Muka T, Ruiter R, de Keyser CE, Hofman A, Franco OH, *et al.* Dietary Fiber Intake Modifies the Positive Association between n-3 PUFA Intake and Colorectal Cancer Risk in a Caucasian Population. J Nutr **2015**;145(8):1709-16 doi 10.3945/jn.114.208462.
23. Navarro SL, Neuhouser ML, Cheng TD, Tinker LF, Shikany JM, Snetselaar L, *et al.* The Interaction between Dietary Fiber and Fat and Risk of Colorectal Cancer in the Women's Health Initiative. Nutrients **2016**;8(12) doi 10.3390/nu8120779.
24. Shin A, Cho S, Sandin S, Lof M, Oh MY, Weiderpass E. Omega-3 and -6 Fatty Acid Intake and Colorectal Cancer Risk in Swedish Women's Lifestyle and Health Cohort. Cancer Res Treat **2020** doi 10.4143/crt.2019.550.
25. Schuurman AG, van den Brandt PA, Dorant E, Brants HA, Goldbohm RA. Association of energy and fat intake with prostate carcinoma risk: results from The Netherlands Cohort Study. Cancer **1999**;86(6):1019-27.
26. Mannisto S, Pietinen P, Virtanen MJ, Salminen I, Albanes D, Giovannucci E, *et al.* Fatty acids and risk of prostate cancer

in a nested case-control study in male smokers. Cancer Epidemiol Biomarkers Prev **2003**;12(12):1422-8.
27. Laaksonen DE, Laukkanen JA, Niskanen L, Nyyssonen K, Rissanen TH, Voutilainen S, *et al.* Serum linoleic and total polyunsaturated fatty acids in relation to prostate and other cancers: a population-based cohort study. Int J Cancer **2004**;111(3):444-50 doi 10.1002/ijc.11614.
28. Leitzmann MF, Stampfer MJ, Michaud DS, Augustsson K, Colditz GC, Willett WC, *et al.* Dietary intake of n-3 and n-6 fatty acids and the risk of prostate cancer. Am J Clin Nutr **2004**;80(1):204-16 doi 10.1093/ajcn/80.1.204.
29. Neuhouser ML, Barnett MJ, Kristal AR, Ambrosone CB, King I, Thornquist M, *et al.* (n-6) PUFA increase and dairy foods decrease prostate cancer risk in heavy smokers. J Nutr **2007**;137(7):1821-7 doi 10.1093/jn/137.7.1821.
30. Park SY, Murphy SP, Wilkens LR, Henderson BE, Kolonel LN. Fat and meat intake and prostate cancer risk: the multiethnic cohort study. Int J Cancer **2007**;121(6):1339-45 doi 10.1002/ijc.22805.
31. Wallstrom P, Bjartell A, Gullberg B, Olsson H, Wirfalt E. A prospective study on dietary fat and incidence of prostate cancer (Malmo, Sweden). Cancer Causes Control **2007**;18(10):1107-21 doi 10.1007/s10552-007-9050-4.
32. Bassett JK, Severi G, Hodge AM, MacInnis RJ, Gibson RA, Hopper JL, *et al.* Plasma phospholipid fatty acids, dietary fatty acids and prostate cancer risk. Int J Cancer **2013**;133(8):1882-91 doi 10.1002/ijc.28203.
33. Pelser C, Mondul AM, Hollenbeck AR, Park Y. Dietary fat, fatty acids, and risk of prostate cancer in the NIH-AARP diet and health study. Cancer Epidemiol Biomarkers Prev **2013**;22(4):697-707 doi 10.1158/1055-9965.epi-12-1196-t.
34. Stolzenberg-Solomon RZ, Pietinen P, Taylor PR, Virtamo J, Albanes D. Prospective study of diet and pancreatic cancer in male smokers. Am J Epidemiol **2002**;155(9):783-92 doi 10.1093/aje/155.9.783.
35. Michaud DS, Giovannucci E, Willett WC, Colditz GA, Fuchs CS. Dietary meat, dairy products, fat, and cholesterol and pancreatic cancer risk in a prospective study. Am J Epidemiol **2003**;157(12):1115-25 doi 10.1093/aje/kwg098.
36. Heinen MM, Verhage BA, Goldbohm RA, van den Brandt PA. Meat and fat intake and pancreatic cancer risk in the Netherlands Cohort Study. Int J Cancer **2009**;125(5):1118-26 doi 10.1002/ijc.24387.
37. Thiebaut AC, Jiao L, Silverman DT, Cross AJ, Thompson FE, Subar AF, *et al.* Dietary fatty acids and pancreatic cancer in the NIH-AARP diet and health study. J Natl Cancer Inst **2009**;101(14):1001-11 doi 10.1093/jnci/djp168.
38. Jain MG, Rohan TE, Howe GR, Miller AB. A cohort study of nutritional factors and endometrial cancer. Eur J Epidemiol **2000**;16(10):899-905.
39. Brasky TM, Rodabough RJ, Liu J, Kurta ML, Wise LA, Orchard TS, *et al.* Long-chain omega-3 fatty acid intake and endometrial cancer risk in the Women's Health Initiative. Am J Clin Nutr **2015**;101(4):824-34 doi 10.3945/ajcn.114.098988.
40. Brasky TM, Sponholtz TR, Palmer JR, Rosenberg L, Ruiz-Narvaez EA, Wise LA. Associations of Dietary Long-Chain omega-3 Polyunsaturated Fatty Acids and Fish Consumption With Endometrial Cancer Risk in the Black Women's Health Study. Am J Epidemiol **2016**;183(3):199-209 doi 10.1093/aje/kwv231.
41. Bertone ER, Rosner BA, Hunter DJ, Stampfer MJ, Speizer FE, Colditz GA, *et al.* Dietary fat intake and ovarian cancer in a cohort of US women. Am J Epidemiol **2002**;156(1):22-31 doi 10.1093/aje/kwf008.
42. Wallingford SC, van As JA, Hughes MC, Ibiebele TI, Green AC, van der Pols JC. Intake of omega-3 and omega-6 fatty acids and risk of basal and squamous cell carcinomas of the skin: a longitudinal community-based study in Australian adults. Nutr Cancer **2012**;64(7):982-90 doi 10.1080/01635581.2012.713540.
43. Park MK, Li WQ, Qureshi AA, Cho E. Fat Intake and Risk of Skin Cancer in U.S. Adults. Cancer Epidemiol Biomarkers Prev **2018**;27(7):776-82 doi 10.1158/1055-9965.epi-17-0782.
44. Luu HN, Cai H, Murff HJ, Xiang YB, Cai Q, Li H, *et al.* A prospective study of dietary polyunsaturated fatty acids intake and lung cancer risk. Int J Cancer **2018**;143(9):2225-37 doi 10.1002/ijc.31608.
45. Koh WP, Dan YY, Goh GB, Jin A, Wang R, Yuan JM. Dietary fatty acids and risk of hepatocellular carcinoma in the Singapore Chinese health study. Liver Int **2016**;36(6):893-901 doi 10.1111/liv.12978.
46. Yang W, Sui J, Ma Y, Simon TG, Petrick JL, Lai M, *et al.* High Dietary Intake of Vegetable or Polyunsaturated Fats is Associated With Reduced Risk of Hepatocellular Carcinoma. Clin Gastroenterol Hepatol **2020** doi 10.1016/j.cgh.2020.01.003.
47. Chajes V, Hulten K, Van Kappel AL, Winkvist A, Kaaks R, Hallmans G, *et al.* Fatty-acid composition in serum phospholipids and risk of breast cancer: an incident case-control study in Sweden. Int J Cancer **1999**;83(5):585-90 doi 10.1002/(sici)1097-0215(19991126)83:5<585::aid-ijc2>3.0.co;2-z.
48. Pala V, Krogh V, Muti P, Chajes V, Riboli E, Micheli A, *et al.* Erythrocyte membrane fatty acids and subsequent breast cancer: a prospective Italian study. J Natl Cancer Inst **2001**;93(14):1088-95 doi 10.1093/jnci/93.14.1088.
49. Saadatian-Elahi M, Toniolo P, Ferrari P, Goudable J, Akhmedkhanov A, Zeleniuch-Jacquotte A, *et al.* Serum fatty acids and risk of breast cancer in a nested case-control study of the New York University Women's Health Study. Cancer Epidemiol Biomarkers Prev **2002**;11(11):1353-60.
50. Rissanen H, Knekt P, Jarvinen R, Salminen I, Hakulinen T. Serum fatty acids and breast cancer incidence. Nutr Cancer **2003**;45(2):168-75 doi 10.1207/s15327914nc4502_05.
51. Wirfalt E, Vessby B, Mattisson I, Gullberg B, Olsson H, Berglund G. No relations between breast cancer risk and fatty acids of erythrocyte membranes in postmenopausal women of the Malmo Diet Cancer cohort (Sweden). Eur J Clin Nutr **2004**;58(5):761-70 doi 10.1038/sj.ejcn.1601874.
52. Chajes V, Thiebaut AC, Rotival M, Gauthier E, Maillard V, Boutron-Ruault MC, *et al.* Association between serum trans-monounsaturated fatty acids and breast cancer risk in the E3N-EPIC Study. Am J Epidemiol **2008**;167(11):1312-20 doi 10.1093/aje/kwn069.
53. Takata Y, King IB, Neuhouser ML, Schaffer S, Barnett M, Thornquist M, *et al.* Association of serum phospholipid fatty acids with breast cancer risk among postmenopausal cigarette smokers. Cancer Causes Control **2009**;20(4):497-504 doi 10.1007/s10552-009-9314-2.
54. Pouchieu C, Chajes V, Laporte F, Kesse-Guyot E, Galan P, Hercberg S, *et al.* Prospective associations between plasma saturated, monounsaturated and polyunsaturated fatty acids and overall and breast cancer risk - modulation by antioxidants:

a nested case-control study. PLoS One **2014**;9(2):e90442 doi 10.1371/journal.pone.0090442.
55. Chajes V, Assi N, Biessy C, Ferrari P, Rinaldi S, Slimani N*, et al.* A prospective evaluation of plasma phospholipid fatty acids and breast cancer risk in the EPIC study. Ann Oncol **2017**;28(11):2836-42 doi 10.1093/annonc/mdx482.
56. Hirko KA, Chai B, Spiegelman D, Campos H, Farvid MS, Hankinson SE*, et al.* Erythrocyte membrane fatty acids and breast cancer risk: a prospective analysis in the nurses' health study II. Int J Cancer **2018**;142(6):1116-29 doi 10.1002/ijc.31133.
57. Kojima M, Wakai K, Tokudome S, Suzuki K, Tamakoshi K, Watanabe Y*, et al.* Serum levels of polyunsaturated fatty acids and risk of colorectal cancer: a prospective study. Am J Epidemiol **2005**;161(5):462-71 doi 10.1093/aje/kwi066.
58. Hall MN, Campos H, Li H, Sesso HD, Stampfer MJ, Willett WC*, et al.* Blood levels of long-chain polyunsaturated fatty acids, aspirin, and the risk of colorectal cancer. Cancer Epidemiol Biomarkers Prev **2007**;16(2):314-21 doi 10.1158/1055-9965.epi-06-0346.
59. Butler LM, Yuan JM, Huang JY, Su J, Wang R, Koh WP*, et al.* Plasma fatty acids and risk of colon and rectal cancers in the Singapore Chinese Health Study. NPJ Precis Oncol **2017**;1(1):38 doi 10.1038/s41698-017-0040-z.
60. Harvei S, Bjerve KS, Tretli S, Jellum E, Robsahm TE, Vatten L. Prediagnostic level of fatty acids in serum phospholipids: omega-3 and omega-6 fatty acids and the risk of prostate cancer. Int J Cancer **1997**;71(4):545-51 doi 10.1002/(sici)1097-0215(19970516)71:4<545::aid-ijc7>3.0.co;2-u.
61. Chavarro JE, Stampfer MJ, Li H, Campos H, Kurth T, Ma J. A prospective study of polyunsaturated fatty acid levels in blood and prostate cancer risk. Cancer Epidemiol Biomarkers Prev **2007**;16(7):1364-70 doi 10.1158/1055-9965.epi-06-1033.
62. Crowe FL, Allen NE, Appleby PN, Overvad K, Aardestrup IV, Johnsen NF*, et al.* Fatty acid composition of plasma phospholipids and risk of prostate cancer in a case-control analysis nested within the European Prospective Investigation into Cancer and Nutrition. Am J Clin Nutr **2008**;88(5):1353-63 doi 10.3945/ajcn.2008.26369.
63. Park SY, Wilkens LR, Henning SM, Le Marchand L, Gao K, Goodman MT*, et al.* Circulating fatty acids and prostate cancer risk in a nested case-control study: the Multiethnic Cohort. Cancer Causes Control **2009**;20(2):211-23 doi 10.1007/s10552-008-9236-4.
64. Brasky TM, Till C, White E, Neuhouser ML, Song X, Goodman P*, et al.* Serum phospholipid fatty acids and prostate cancer risk: results from the prostate cancer prevention trial. Am J Epidemiol **2011**;173(12):1429-39 doi 10.1093/aje/kwr027.
65. Brasky TM, Darke AK, Song X, Tangen CM, Goodman PJ, Thompson IM*, et al.* Plasma phospholipid fatty acids and prostate cancer risk in the SELECT trial. J Natl Cancer Inst **2013**;105(15):1132-41 doi 10.1093/jnci/djt174.
66. Cheng TY, King IB, Barnett MJ, Ambrosone CB, Thornquist MD, Goodman GE*, et al.* Serum phospholipid fatty acids, genetic variation in myeloperoxidase, and prostate cancer risk in heavy smokers: a gene-nutrient interaction in the carotene and retinol efficacy trial. Am J Epidemiol **2013**;177(10):1106-17 doi 10.1093/aje/kws356.
67. Matejcic M, Lesueur F, Biessy C, Renault AL, Mebirouk N, Yammine S*, et al.* Circulating plasma phospholipid fatty acids and risk of pancreatic cancer in a large European cohort. Int J Cancer **2018**;143(10):2437-48 doi 10.1002/ijc.31797.
68. Wallingford SC, Hughes MC, Green AC, van der Pols JC. Plasma omega-3 and omega-6 concentrations and risk of cutaneous basal and squamous cell carcinomas in Australian adults. Cancer Epidemiol Biomarkers Prev **2013**;22(10):1900-5 doi 10.1158/1055-9965.EPI-13-0434.
69. Morimoto Y, Conroy SM, Ollberding NJ, Henning SM, Franke AA, Wilkens LR*, et al.* Erythrocyte membrane fatty acid composition, serum lipids, and non-Hodgkin's lymphoma risk in a nested case-control study: the multiethnic cohort. Cancer Causes Control **2012**;23(10):1693-703 doi 10.1007/s10552-012-0048-1.
70. Chajes V, Jenab M, Romieu I, Ferrari P, Dahm CC, Overvad K*, et al.* Plasma phospholipid fatty acid concentrations and risk of gastric adenocarcinomas in the European Prospective Investigation into Cancer and Nutrition (EPIC-EURGAST). Am J Clin Nutr **2011**;94(5):1304-13 doi 10.3945/ajcn.110.005892.
